# Supplementary material for: Complexome profiling of the Chlamydomonas psb28 mutant reveals TEF5 as an early PSII assembly factor
Source: Plant Cell. 2025 Mar 17;37(6):koaf055. doi: 10.1093/plcell/koaf055 (PMC12164590; doi:10.1093/plcell/koaf055)
Supplement: koaf055_Supplementary_Data [file koaf055_supplementary_data.zip › psb28_interactive.zip!/plots/intensity/qualityPlots/peptides/PLAP1_Cre01.g039550.t1.1.html]

### Cre01.g039550.t1.1

Trivial name: PLAP1  
SeqCoverage [median]: 2.40  
Mass: 175.74 kDa  
  
 

| Sequence | MissedCleavage | Mass [Da] | wtIntensity | muIntensity | Score |
| --- | --- | --- | --- | --- | --- |
| IALVFEDAR | 0 | 1032.56 | 396.14 | 1456.22 | 74.13 |
| TAAQLASQAVTR | 0 | 1215.66 | 0.00 | 1391.62 | 170.40 |
| TPASSLVFTVGAR | 0 | 1304.71 | 0.00 | 1058.59 | 99.28 |
|  |  |  | 396.1 | 3906.4 |  |
